# A Platform for Specific Delivery of Lanthanide–Scandium Mixed-Metal Cluster Fullerenes into Target Cells

**DOI:** 10.1002/open.201200023

**Published:** 2012-09-28

**Authors:** Anna Svitova, Klaus Braun, Alexey A Popov, Lothar Dunsch

**Affiliations:** a Department of Electrochemistry and Conducting Polymers, Leibniz Institute of Solid State and Material ResearchHelmholtzstrasse 20, 01069 Dresden (Germany) E-mail: L.Dunsch@ifw-dresden.de; b Department of Medical Physics in Radiology, German Cancer Research CenterINF 280, 69120 Heidelberg (Germany) E-mail: k.braun@dkfz.de

**Keywords:** bioshuttles, gadolinium, molecular imaging, nitride cluster fullerenes

Lanthanides (Ln) find broad applications as contrast agents in medical imaging techniques such as magnetic resonance imaging (MRI).[1] MRI is one of the most powerful, noninvasive imaging procedures, which is able to create images of tissues, organs and diseases in vivo.[2, 3] Every year, about six million patients undergo MRI studies of which 30 % are performed using Gd-based contrast agents (CAs), which significantly reduce the spin-lattice relaxation time *T*_1_ of water protons leading to an increase of the signal intensity and improved contrast.[4] Although medical applications of lanthanides in imaging are dominated by Gd^3+^, other lanthanides can be also used as MRI[1, 5] CAs (e.g., Dy^3+^ is considered as an efficient contrast agent for high-field MRI[6]) and X-ray CAs.[7, 8] Radioisotopes of lanthanides (especially ^177^Lu) are employed as therapeutic radiopharmaceuticals.[9]

Direct administration of Ln^3+^ ions in vivo is not possible because of their toxicity in the free ion form.[10] In MRI, organic chelates of Gd^3+^ and other lanthanides are used to circumvent this problem.[3, 11] The toxicity of Gd^3+^ is thus substantially decreased and its solubility in biological fluids is improved, albeit some negative phenomena still remain (e.g., fibrosis effects in kidneys[12]). The encapsulation of Ln^3+^ ions into the hollow carbon cages with formation of endohedral metallofullerenes (Ln-EMF) might be a more advantageous solution for a CAs,[13] because (1) carbon cages protect Ln^3+^ ions against external chemical exposure and their release into the body, and (2) the water proton relaxivity *r*_1_ (the effect on 1/*T*_1_) of Ln-EMFs and especially Gd-EMFs is remarkably higher compared with organic chelates.[14–16] Among the other potential medical applications of Ln-EMFs, their use as X-ray CAs[8] or radiolabeling of Ln-EMFs for imaging and therapy can be mentioned.[17, 18] Combination of different lanthanides, for example, Gd/Lu or Ho/Lu, in one EMF can be used for a design of multimodal contrast media.[19]

The distribution of standard CAs is usually restricted to the blood stream and the interstitial space. As a result, even contrast-enhanced imaging techniques still suffer from insufficient image resolution of morphological structures. The diagnostic problems, such as the limited possibility to exactly determine the actual tumor size and volume, to secure the metastases existence, and to distinguish the tumor tissues from healthy ones, have dramatic consequences for surgery and radiation therapy. The development of “molecular imaging” (MI) as an academic discipline has opened the way for further development in diagnostic imaging procedures.[20] MI can be defined as imaging measurement of the cellular processes on the molecular level.[21] The strategy is based on the targeting of specific proteins, in particular cells or cell parts, and coupling of this targeting with imaging techniques, which can be a promising way to enhance the contrast to differentiate between the tissues.

Reported applications of Ln-EMFs for imaging are mostly limited to the use of their water-soluble derivatives as standard nonspecific MRI agents. Their use for MI-related techniques is very rare, however available results show high potential of Ln-EMFs in MI.[15, 16, 18] For instance, in vitro studies of a GdSc_2_N@C_80_-based BioShuttle system specifically designed to target human MDA-MB-231 breast adenocarcinoma cells showed that at a concentration of only 1/20 of the typical clinical dose, the sensitivity of this system was more than 500-fold higher than that of the commercial MRI-CA, Gd-DTPA (Gd-based complex with diethylene triamine pentaacetic acid), while a cell viability assay did not reveal any cell toxicity of the BioShuttle system.[15]

In this work, we introduce a Ln-EMF-based BioShuttle[22, 23] system as a platform for intracellular delivery of Ln-EMFs into *c-myc* mRNA-expressing cells suitable as an MI and potentially a therapeutic agent. In particular, we describe a synthesis of MI probe c-myc-antisense-Gd@BioShuttle comprised of (1) a Gd-containing nitride cluster fullerene as an imaging component, (2) an address module (nuclear localization sequence), and (3) a transmembrane carrier peptide. Facile transport of this system into the target cells is demonstrated by in vitro studies.

Gd-containing mixed-metal cluster fullerenes were produced by the Krätschmer–Huffman method modified in our group.[24] In this work, we used melamine (C_3_H_6_N_6_; organic base with high nitrogen content of 66 % by mass) as a new selective nitrogen source. The graphite rods packed with a Gd/Sc/graphite/melamine mixture were evaporated in 200 mbar helium atmosphere with a current of 100 A. MS data analysis of the fullerene extract showed formation of EMFs with the mixed-metal nitride clusters with the general formula Gd_*x*_Sc_*3−x*_N@C_*2n*_ (*x*= 0–3, 39≤*n*≤44). Isolation of these mixed-metal nitride cluster fullerenes (NCFs) was accomplished by one-step HPLC. The analysis of the chromatogram and mass spectra proved the formation of NCFs as the major products of the reaction (Figure 1), which demonstrates the high selectivity of the synthesis using melamine as a nitrogen source (recently, similar selectivity was reported for urea[25]). The most abundant fraction eluting at a retention time (*t*_R_) of 29.8–32.0 min was collected and used for further synthesis of the modular contrast agent c-myc-antisense-Gd@BioShuttle (Figure 1 A). MS data analysis revealed that this fraction mainly consists of GdSc_2_N@C_80_ (I), Sc_3_N@C_78_, and Gd_2_ScN@C_80_ (I) (the ratio of the total ion currents corresponding to each molecule is 4:1.3:1, respectively), although trace amounts of Sc_3_N@C_80_, Sc_4_C_2_@C_80_ and GdSc_2_N@C_78_ are also seen in the mass spectrum (Figure 1 B). The relative yield of this fraction determined from the area of the HPLC peaks is 63 % of all endohedral fullerenes.

**Figure 1:**
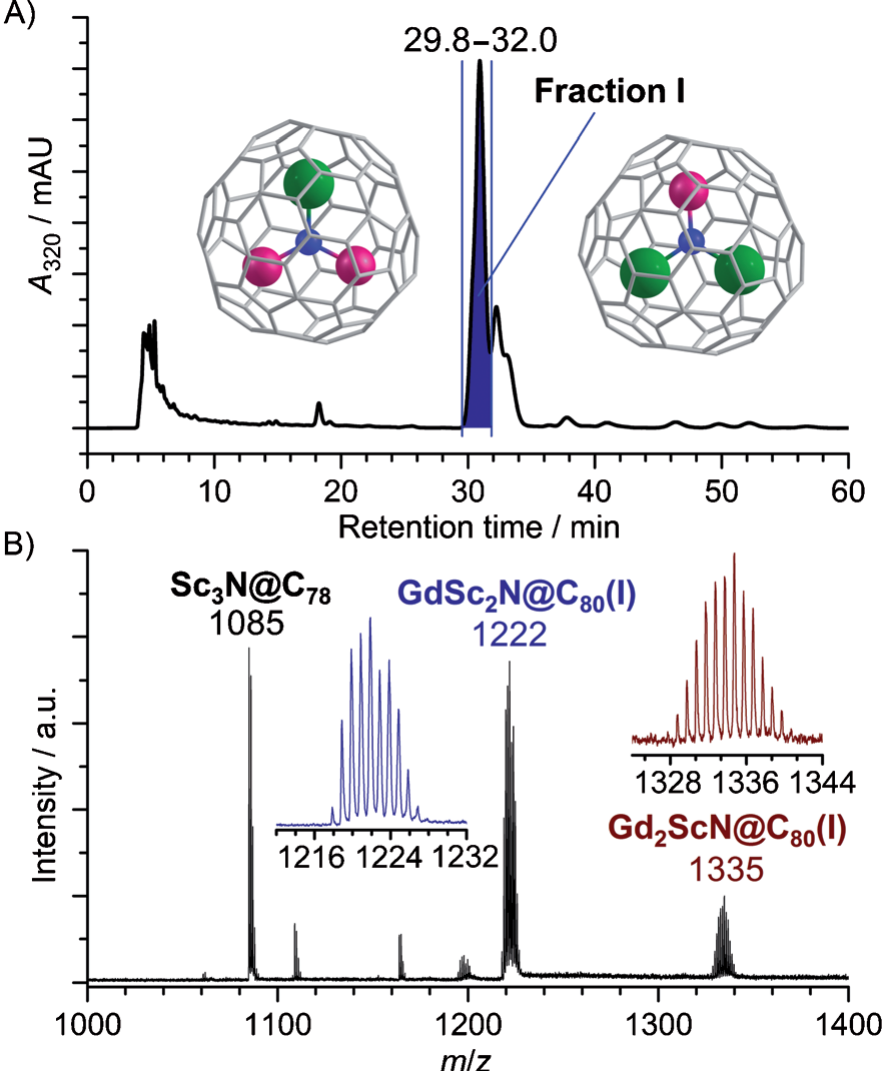
A) Chromatogram of Gd_x_Sc_3−x_N@C_2n_ fullerene extract mixture synthesized by the arc burning method (combination of two 4.6×250 mm Buckyprep columns; flow rate of 1.6 mL min^−1^; toluene as eluent; 40 °C), molecular structures of GdSc_2_N@C_80_ (I) and Gd_2_ScN@C_80_ (I) are shown. B) Positive-ion MALDI-TOF MS of the major HPLC fraction collected at *t*_R_=29.8–32.0 min; insets show the isotopic patterns of GdSc_2_N@C_80_ and Gd_2_ScN@C_80_.

The structure of the c-myc-antisense-Gd@BioShuttle system is depicted in Figure 2 (detailed procedures for the synthesis of *c-myc* mRNA-targeted BioShuttle system and for conjugating the BioShuttle with Gd-EMF were described earlier,[15, 22, 23, 26] and are briefly described in the Experimental Section). The BioShuttle complex consists of three functional components. The first component is a transport module consisting of a cell-penetrating peptide (CPP), an amphiphilic molecule responsible for the delivery of previously non-transportable molecules through biological membranes. This first part is connected via a disulfide bridge to a second part, which is the address component of the system. The address module comprises a peptide nucleic acid (PNA) directed against *c-myc* mRNA-expressing cells. *C-myc* mRNA is a template for the Myc protein, which is implicated in the rapid growth of cancer cells and is barely present in normal cells. In all *c-myc*-expressing cells, the PNA antisense sequence hybridizes with the *c-myc* mRNA (in the vicinity of *c-myc* exon II) providing high cell specificity. The hybrid remains in the cytoplasm of targeted cells together with the cargo module, which in this case is GdSc_2_N@C_80_ connected via a bridge to the address module. Thus, by its design, the BioShuttle system has high specificity, because of the trapping of the gadolinium fullerene as an MRI component into the neoplastic cells with an aberrant gene expression profile in contrast to normal cells that do not reveal hybridization possibilities.

**Figure 2:**
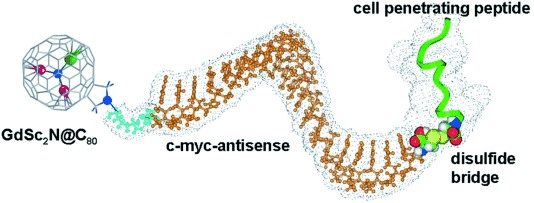
Modular structure of the c-myc-antisense-Gd@BioShuttle conjugate: The module cell-penetrating peptide (CPP) (green) is connected via the cleavable disulfide bridge (yellow) to the address module (c-myc antisense PNA; exon II) (ocher), which in turn is ligated covalently with the imaging component as a cargo via a lysine spacer (blue). The fluorescence dye is coupled to the ε-amino group of the lysine.

To visualize the transport of the c-myc-antisense-Gd@BioShuttle system across biological membranes and its intracellular localization, confocal laser scanning microscopy (CLSM) measurements were performed. To perform CLSM studies, DU145 human prostate cancer cells were incubated with the c-myc-antisense-Gd@BioShuttle complex labeled with an Alexa Fluor® 546-conjugated fluorescent dye at the non-cleavable lysine-spacer site on the ε-amino group.[22] In Figure 3, the intracellular localization of the c-myc-antisense-Gd@BioShuttle transporter after 5, 10, 20 and 30 min incubation time is illustrated via clear cytoplasmic fluorescence signals. Excitation was carried out at 543 nm (He/Ne laser), and the emission wavelength range was 572–650 nm.

**Figure 3:**
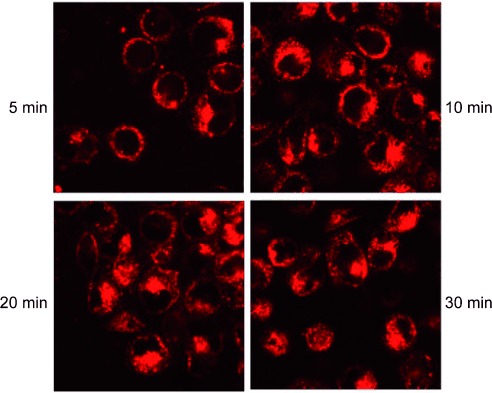
CLSM study of the transport of Alexa Fluor® 546-labeled c-myc- antisense Gd@BioShuttle into *c-myc* mRNA-positive human prostate cancer cells DU145. Incubation time was increased from 5 to 30 min. Excitation wavelength: 543 nm; emission detection window: 572–650 nm.

In the first five minutes after incubation, the CLSM measurements showed a rapid accumulation of perinuclear fluorescence signal near the cell membrane, which gradually changed into a cytoplasm-localized signal after 30 min. This proves that the CA was transported into the cells with an aberrant *c-myc* gene expression profile. In contrast to the *c-myc* expressing cancer cells, no signal is detectable in normal cells because of the lack of mRNA/PNA hybridization (data not shown). Thus, the CLSM study demonstrates an effective transport of c-myc-antisense-Gd@BioShuttle complexes in human cancer cells showing the possibility of using this complex as an intracellular CA appropriate for an MI approach. It also can act as a suitable carrier system for diagnostic and therapeutic agents in cancer therapy.

In conclusion, Gd-containing nitride cluster fullerenes were synthesized as the main fullerene products at high relative yield using direct current (dc) arc discharge as the method and melamine as a new solid source of nitrogen. The mixed-metal cluster fullerenes Gd_*x*_Sc_*3−x*_N@C_*2n*_ were introduced as a paramagnetic imaging module into the new c-myc-antisense-Gd@BioShuttle system. Facile intracellular transport and high specificity for cells that have an aberrant gene expression of a dye-labeled system was demonstrated by fluorescence spectroscopy. Thus, the Ln-EMF-BioShuttle concept can be used as a new molecular-imaging system with promising diagnostic and therapeutic medical applications.

## Experimental Section

**Synthesis of fullerenes**: The Krätschmer-Huffman synthesis was used. The graphite rods (length 100 mm, diameter 8 mm) were drilled, and the holes filled with a mixture gadolinium and scandium, graphite powder and melamine (C_3_H_6_N_6_) in the optimized ratio of Gd/Sc/C/N=1:1:15. The arc-discharge reactor for fullerene production consists of a water-cooled cylindrical chamber with two holders for graphite rods. A current of approx. 100 A was applied to evaporate the packed graphite rods in a 200 mbar helium atmosphere. The collected soot was first pre-extracted with acetone for 1 h to remove non-fullerene products like polycyclic aromatic hydrocarbons (PAH) and other low molecular structures. The fullerene mixture was then Soxhlet extracted by CS_2_ for 20 h. After the removal of CS_2_, the sample was redissolved in toluene. The isolation of mixed-metal nitride cluster fullerenes was accomplished by one-step HPLC using analytical Buckyprep columns (4.6×250 mm; Nacalai Tesque, Japan). The fraction eluting at *t*_R_=29.8–32.0 min was collected and subsequently characterized by MALDI-TOF MS with a Biflex III spectrometer (Bruker, Germany).

**Synthesis of c-myc-antisense-Gd@BioShuttle system**: The details of the synthetic procedures of the BioShuttle system were described earlier.[23, 27] Briefly, the acid chloride of the fullerene was obtained by the method of Arrowsmith et al.[28] For the synthesis of *N*-Boc-propyldiamin-tetrazin-dien, the acid chloride was suspended in abs CH_2_Cl_2_ (20 mL), and a mixture of *N*-Boc-1,3-diaminopropane (2 mmol) and Et_3_N (2 mmol) in the same solvent (10 mL) was added at 0–5 °C. The resulting solution was maintained at RT for 4 h, and the organic phase was washed with H_2_O, followed by 1 n HCl, and then again with H_2_O. The organic layer was dried over Na_2_SO_4_, filtered, and evaporated. The resulting residue was purified by column chromatography (silica gel, CHCl_3_/EtOH, 9:1). Sequences of single modules as well as the complete modular construct were purified by analytical HPLC (Shimadzu LC-8A, Duisburg, Germany) on a YMC ODS-A 7A S 7 µm reverse-phase column (20×250 mm). The fullerene(aminobonded)-tetrazoline-diene was obtained as follows. Monosubstituted tetrazinamine (0.5 mmol) and 4-methyl-5-oxo-2,3,4,6,8-pentazabicyclo[4.3.0]nona-2,7,9-trien-9-carboxylic acid chloride (0.5 mmol) were dissolved in CHCl_3_/Et_3_N (1:1, *v*/*v*) for 4 h at 0–5 °C. The solution was washed with H_2_O, followed by 1 n HCl, and then again with H_2_O. The organic layer was dried over Na_2_SO_4_, filtered, and evaporated. The residue was finally purified by HPLC (silica gel, CHCl_3_/EtOH, 9.5/0.5).

To perform the solid-phase peptide synthesis (SPPS) of peptide modules, we used a strategy described by Merrifield[29] and Carpino,[30] employing a fully automated synthesizer Syro II (MultiSyn Tech, Germany). The c-myc-antisense-Gd@BioShuttle conjugates were labeled with Alexa Fluor® 546 at the non-cleavable lysine-spacer site on the ε-amino group.

Cysteine groups of the cell-penetrating peptide (CPP) (transport module) and the peptide nucleic acid (PNA) (address module) with imaging component were oxidized at the range of 2 mg mL^−1^ in a 20 % DMSO/H_2_O solution, with the reaction reaching completion after 5 h. The progress of oxidation was monitored by analytical C18 reverse-phase HPLC.

**Confocal laser scanning microscopy (CLSM) measurements**: We used DU145 human prostate cancer cells that were characterized by Stone et al.[31] The procedure of cell preparation was described earlier.[32] Briefly, the cells were maintained in RPMI1640 medium (Gibco 11825) supplemented with fetal calf serum (FCS; 2 %; Gibco). The final BioShuttle concentration was 100 nM, and physiologic NaCl solution was used as a solvent. The studies and the control experiments were accomplished under identical conditions as detailed by Braun et al.[22]
